# The Antitumor Effect of Gekko Sulfated Glycopeptide by Inhibiting bFGF-Induced Lymphangiogenesis

**DOI:** 10.1155/2016/7396392

**Published:** 2016-04-12

**Authors:** Xiu-Li Ding, Ya-Nan Man, Jian Hao, Cui-Hong Zhu, Chang Liu, Xue Yang, Xiong-Zhi Wu

**Affiliations:** ^1^Zhong-Shan-Men In-Patient Department, Tianjin Medical University Cancer Institute and Hospital, National Clinical Research Center for Cancer, Key Laboratory of Cancer Prevention and Therapy, Huan-Hu-Xi Road, He-Xi District, Tianjin 300060, China; ^2^Department of Radiotherapy, The Second Affiliated Hospital of Tianjin Medical University, Ping-Jiang Road, He-Xi District, Tianjin 300060, China

## Abstract

*Objective*. To study the antilymphangiogenesis effect of Gekko Sulfated Glycopeptide (GSPP) on human lymphatic endothelial cells (hLECs).* Methods*. MTS was conducted to confirm the antiproliferation effect of GSPP on hLECs; flow cytometry was employed to detect hLECs cycle distribution; the antimigration effect of GSPP on hLECs was investigated by wound healing experiment and transwell experiment; tube formation assay was used to examine its inhibitory effect on the lymphangiogenesis; western blotting was conducted to detect the expression of extracellular signal-regulated kinase1/2 (Erk1/2) and p-Erk1/2 after GSPP and basic fibroblast growth factor (bFGF) treatment. Nude mice models were established to investigate the antitumor effect of GSPP in vivo. Decreased lymphangiogenesis caused by GSPP in vivo was verified by immunohistochemical staining.* Results*. In vitro, GSPP (10 *μ*g/mL, 100 *μ*g/mL) significantly inhibited bFGF-induced hLECs proliferation, migration, and tube-like structure formation (*P* < 0.05) and antagonized the phosphorylation activation of Erk1/2 induced by bFGF. In vivo, GSPP treatment (200 mg/kg/d) not only inhibited the growth of colon carcinoma, but also inhibited the tumor lymphangiogenesis.* Conclusion*. GSPP possesses the antitumor ability by inhibiting bFGF-inducing lymphangiogenesis in vitro and in vivo, which may further inhibit tumor lymphatic metastasis.

## 1. Introduction

There are two vital ways for cancer cells to disseminate: the blood pathway, leading to the invasion of distant organs such as liver, brain, bone, or lung, and the lymphatic pathway, leading to the invasion of the lymph nodes draining the organs where the tumor evolves [1]. For a long time, the study on tumor metastasis has been centered on tumor angiogenesis and hematogenous metastasis [2]. In the recent years, tumor lymphangiogenesis catches the eyes of researchers [3]. One of the most popular views on cancerous lymphangiogenesis is that tumor forms new lymphatic vessels on the basis of the existing ones. Studies have demonstrated that tumor-induced lymphangiogenesis plays a significant role in tumor cells traffic and lymph node metastasis [4–6]. Further, lymphatic metastasis is a major and early step during tumor progression. The presence of lymphatic metastasis is a key determinant of cancer staging, treatment, and prognosis [7, 8]. Thus, antilymphangiogenesis is a new target for cancer therapy. However, there are no effective antilymphangiogenesis drugs in clinic until now.

It was reported that vascular endothelial growth factor-C3 (VEGF-C3) and VEGF-D were identified as the stimulators of the proliferation of lymphatic endothelial cells. Recent studies showed that basic fibroblast growth factor (bFGF) was another effective stimulator for lymphangiogenesis and could regulate lymphatic endothelial cell proliferation, migration, and tube formation as well [9–11]. bFGF is a heparin dependent growth factor and can activate the intracellular signal transduction pathways only in the form of bFGF-heparin-FGFR terpolymers structure [12]. bFGF is bound to heparan sulfate (HS) in the extracellular matrix (ECM) and is released in an active form when the ECM-HS is degraded by heparanase expressed in normal and malignant cells [13, 14].

We previously isolated a novel polysaccharide, Gekko Sulfated Glycopeptide (GSPP) from* Gekko swinhonis *Günther, and confirmed it as a homogeneous sulfated polysaccharide-protein complex with O-glycopeptide linkages. The molecular weight of GSPP was estimated to be over 2000 kDa [15]. Its direct effects on the proliferation, differentiation, and migration of hepatoma cells have been studied [15–17]. Our further study showed that GSPP had a similar structure with heparin and competed with heparin to disturb the bFGF-heparin-FGFR terpolymers forming, further blocking bFGF's biological effect. GSPP could inhibit tumor angiogenesis by reducing bFGF production, inhibiting the release of bFGF from the extracellular matrix, and disturbing the binding of bFGF to its low affinity receptor. By inhibiting bFGF-induced angiogenesis, GSPP significantly inhibited the growth of nude mice xenografted tumors [12].

In this study, whether GSPP could inhibit the lymphangiogenesis goaded our interests. Here, we investigated the potent antilymphangiogenesis ability of GSPP in vitro and in vivo and found that GSPP significantly inhibited bFGF-induced cell proliferation, migration, and tube formation in hLECs. And GSPP demonstrated an excellent antitumor effect through inhibiting lymphangiogenesis in vivo.

## 2. Materials and Methods

### 2.1. Cell Lines

hLECs were purchased from CHI Scientific Inc. (Jiangsu, China). The certificate analysis sheet supplied by CHI Scientific Inc. for each vial of cells indicated that more than 95% of the cells were hLECs (CD31 and podoplanin double positive). This was determined by Fluorescence Activating Cell Sorter (FACS). Cells were cultured in EGM-2 media according to the supplier's instructions (CHI Scientific Inc., Jiangsu, China). Cells before 6 generations were used in this study.

### 2.2. Antibodies and Reagents

GSPP used in this study was prepared in advance, which is the same batch with that in previous study [15]. The dried powder is stored in −80°C. MTS test kit was purchased from Promega Corporation (Madison, Wisconsin, USA). Fibronectin from human plasma was purchased from Sigma (St. Louis, MO, USA). Recombinant human FGF-basic (154 a.a.) was purchased from PeproTech Corporation (Rocky Hill, NJ, USA). Antibodies against phospho-Erk1/2 (p44/42 MAPK, lot: 9101S) and total-Erk1/2 (p44/42 MAPK, 137F5, lot: 4695) were obtained from Cell Signaling Technology (Danvers, MA, USA). Antibody against LYVE-1 (lot: 33504-1) was bought from Abcam Corporation (Cambridge, MA, USA). Antibody against *β*-actin (lot: A2228) was bought from Sigma Corporation (St. Louis, MO, USA).

### 2.3. hLECs Proliferation Assay

96-well plate was precoated with fibronectin for 20 minutes at 37°C in 5% humidified CO_2_. 100 *μ*L hLECs suspension (1 × 10^5^ cells/mL) was seeded into each well of a 96-well plate. After 24 h, the medium was discarded and replaced with drug-containing medium. 6 groups were set up as GSPP 10 *µ*g/mL, GSPP 100 *µ*g/mL, bFGF 10 ng/mL, GSPP 10 *µ*g/mL with bFGF 10 ng/mL, GSPP 100 *µ*g/mL with bFGF 10 ng/mL, and negative control group. After cells were exposed to the drugs for indicated times (0, 1, 2, 3, 4, 5, and 6 d), 20 *μ*L MTS solution reagent was added into each well and incubated at 37°C for 1–4 h and then the OD value was measured with a Microplate reader (iMark, Bio-Rad) at 490 nm. The media were not changed during the treatment period.

### 2.4. Cell Cycle Detection

A flow cytometry (BD Biosciences, Franklin Lakes, NJ, USA) was used to evaluate cell cycle distribution. hLECs (5 × 10^5^ cells/2 mL) were seeded into 6-well plates and treated with GSPP (10 *µ*g/mL, 100 *µ*g/mL) alone or combined with bFGF 10 ng/mL for 48 h. Cells were collected and washed twice in cold PBS, fixed in 70% methanol (−20°C) overnight. Then, cells were washed with PBS twice again and incubated with RNase (20 *µ*g/mL) in 37°C for 1 h. Propidium iodide (50 *µ*g/mL) was added before being detected by flow cytometry system.

### 2.5. Wound Healing Experiment

hLECs were seeded in 24-well plates at the density of 1 × 10^5^ cells/mL. After cell attachment, hLECs were starved with serum-free EBM-2 for 24 h. A linear wound about 1 mm in width was made by scratching the monolayer cell culture with a pipette tip after cell confluency. Then, EBM-2 with different concentrations of GSPP (10 *µ*g/mL, GSPP 100 *µ*g/mL) and/or bFGF (10 ng/mL) with 15% FBS were added. After 0 and 6 h, the photographs of wound healing width of hLECs were observed and taken under an invert microscope. The migration width was measured by the Photoshop software. The migration ratio was calculated as the migration width of experiment group/the migration width of control group.

### 2.6. Transwell Experiment

After being starved with serum-free medium for 24 h, hLECs (5 × 10^4^ cells) in EBM-2 media with different concentrations of GSPP (10 *µ*g/mL, GSPP 100 *µ*g/mL) were added to the upper chambers of the transwell insert (BD Biosciences, Bedford, MA). EBM-2 containing bFGF was added to the lower chamber to induce cell migration. After being incubated for 12 h at 37°C, cells on the top surface of the membranes were wiped off with cotton balls, and the cells that migrated on the underside of inserts were fixed with methanol and stained with crystal violet. Five different digital images were taken per well, and the numbers of migrated cells were counted and calculated.

### 2.7. Tube Formation Assay

Matrigel was thawed at 4°C overnight. 96-well plate and 100 *μ*L pipette tips were also kept at 4°C overnight and both the plate and tips were placed on ice during the entire experiment process. 30 uL Matrigel was loaded in each well of the 96-well plates and the plate was incubated at 37°C in a tissue culture incubator for 30 min to allow the matrix to polymerize. Trypsinized LECs were adjusted to the appropriate cell density (1.5 × 10^4^ cells/well) with different concentration of GSPP and bFGF as described in the proliferation assay. 100 *μ*L hLECs suspension was added on top of the gel in the 96-well plate. The plate was then incubated at 37°C in a tissue culture incubator and the formation of the capillary-like tubes was observed after 4 h. Then, hLECs were observed under inverted microscope and 9 photographs (×40) were taken per hole. The numbers of matrix form of closed irregular polygon were recorded and calculated.

### 2.8. Western Blot Experiment

For western blot analysis of Erk and p-Erk protein expression, confluent cultures of hLECs in 6-hole pate were homogenized in lysis buffer. The protein concentrations were determined using the BCA Protein Quantitation Kit. Equal amounts of lysate protein were subjected to 10% SDS-polyacrylamide gel electrophoresis. The proteins were transferred onto PVDF membranes for immunoblot analysis. Blocking was performed with 5% nonfat dry milk in 0.1% Tween 20 in TBS, followed by immunoblotting with a polyclonal goat anti-human Erk anti-body, p-Erk antibody, and *β*-actin antibody. Specific binding was detected by the ECL plus Western Blotting Detection System.

### 2.9. In Vivo Nude Mice Model

This study was approved by the Tianjin Cancer Institutional Animal ethics Committee (number 2014044). Animal care and experimental procedures followed the Tianjin Medical University guidelines for the care and use of laboratory animals. 4–6-week-old male BALB/c mice were purchased from LianHe LiHua cooperation. To establish a heterotopic colon carcinoma nude mice model, 2.5 × 10^6^ HT-29 cells in 200 *μ*L PBS were injected into the right flank of BALB/c mice. When tumors grew to 10 mm diameter size, tumors were cut off and cut into 1 mm × 1 mm chips and then transplanted to other 24 BALB/c mice, which were divided into four groups of 8 mice each group. Mice were treated daily with an intraperitoneal injection of either 0.1 mL GSPP (20, 200 mg/kg/day in PBS) or PBS (control) for 21 days. The length and width of the tumors were measured with a caliper every 2-3 days. All mice were sacrificed 24 days after tumor inoculation and the tumors were excised and weighted and the tumor volumes were calculated using the standard formula *V* = *ab* ^2^/2 (*a*, lengths of the tumors; *b*, widths of the tumors).

### 2.10. Immunohistochemistry Staining

Tumor specimens were immediately removed from sacrificed mice and prepared for immunohistological examination. Tumors were fixed in 10% (v/v) neutral buffered formalin overnight, embedded in paraffin and sectioned to a 5 *μ*m thickness. Tumor sections were deparaffinized via immersion in xylene, dehydrated in a graded series of ethanol, and washed with distilled water. Thereafter, tumor sections were boiled in 10 mM sodium citrate buffer (pH = 6.0) for 10 min and cooled at room temperature. To inhibit endogenous peroxidase activity, tumor sections were incubated with methanol containing 1% (v/v) hydrogen peroxide for 10 min. Tumor sections were then blocked with 1% BSA and then incubated overnight with anti-LYVE-1 antibody; tumor sections were probed with peroxidase-conjugated secondary antibodies and incubated with DAB until the desired stain intensity developed. After counterstaining with Harris hematoxylin, tumor sections were examined under an inverted microscope (E100; Nikon, Japan). To analyze immunohistochemical signals within the specimens, all tumor sections were digitized under a ×40 magnification and images were captured.

### 2.11. Statistical Analysis

The data were expressed as the mean ± SD from triplicate experiments and were analyzed using SPSS software (Version 20.0; Chicago, USA). One-way ANOVA and two-way ANOVA were applied to analyze the significance of groups with one factor and two factors, respectively. *P* value <0.05 was considered statistically significant. All of the experiments were repeated at least three times.

## 3. Results

### 3.1. Morphological Characteristics of Lymphatic Endothelial Cells In Vitro Culture

**Figure 1:**
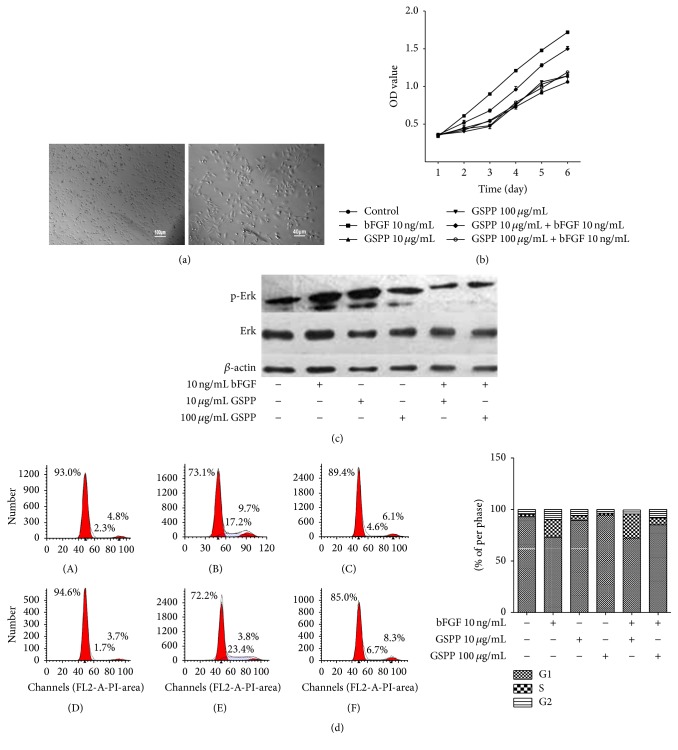
GSPP inhibited bFGF-induced proliferation of hLECs and Erk phosphorylation. (a) Morphological characteristics of hLECs in vitro culture hLECs were compressed ovoid, short fusiform, or polygon and formed a single layer with the characteristic of “pebbles.” (b) hLECs growth curves (1 × 10^5^ cells/mL) were incubated with different concentration of GSPP (0, 10, and 100 *µ*g/mL) and/or bFGF (10 ng/mL) for 0, 1, 2, 3, 4, 5, and 6 d, then cell proliferation was quantified by MTS assay, and cell growth curve was made. (c) The changes of Erk and p-Erk protein expression level of hLECs cultured in 6-well plate were incubated with different concentration of GSPP (0, 10, and 100 *µ*g/mL) and/or bFGF (10 ng/mL); Erk and p-Erk protein levels were monitored by western blot analysis of whole-cell lysates. (d) Cell cycle analysis. Left, histogram of cell cycle distribution. Right, statistical analysis of cell cycle percentage. After exposure to GSPP (0, 10, and 100 *µ*g/mL) and/or bFGF (10 ng/mL) for 48 h, cell cycle distribution was determined by propidium iodide labeling. (A) Control, (B) bFGF 10 ng/mL, (C) GSPP 10 *µ*g/mL, (D) GSPP 100 *µ*g/mL, (E) GSPP 10 *µ*g/mL + bFGF 10 ng/mL, and (F) GSPP 100 *µ*g/mL + bFGF 10 ng/mL. Data were presented as mean ± SD of three independent experiments. hLECs, human lymphatic endothelial cells; bFGF, basic fibroblast growth factor; GSPP, Gekko Sulfated Glycopeptide.

After 3–5 h of primary cell culture, cells were able to grow adhering to the wall. After 1 d, endothelial cells spread forming groups and, after 1 week, cells grew densely and formed a single layer with the characteristic of “pebbles.” Inverted microscopy observation: lymphatic endothelial cells were irregular ovoid with big nucleus and there were many small vacuoles in the cytoplasm (Figure 1(a)).

### 3.2. GSPP Inhibited bFGF-Induced Proliferation of hLECs

MTS assay was performed to investigate the antiproliferation effect of GSPP. After hLECs were exposed to GSPP (0, 10, and 100 *µ*g/mL) and/or bFGF (10 ng/mL) for 0, 1, 2, 3, 4, 5, or 6 d, OD values were detected. The results demonstrated that bFGF promoted the proliferation of hLECs significantly (*P* < 0.05), while GSPP alone did not inhibit proliferation of hLECs (*P* > 0.05). Trypan Blue staining showed that the viability of hLECs was not affected by GSPP (results were not shown). But GSPP could abrogate bFGF-induced proliferation of hLECs significantly in a dose- and time-dependent manner (*P* < 0.05, Figure 1(b)). In the cell cycle assay, bFGF significantly promoted hLECs into proliferation cycle with a high proportion of S and G2 phase cells, while GSPP alone has no effect on the cell cycle distribution of hLECs. When combined with 100 *μ*g/mL GSPP, cell proliferative activity was blocked distinctly with an increasing proportion of G1 phase cells compared with bFGF alone group (Figure 1(d)).

### 3.3. bFGF-Induced p-Erk Was Downregulated by GSPP in hLECs

Erk1/2 signal pathway is reported to be involved in cell growth, migration, and angiogenesis. The promoting effect of bFGF on vessel cell proliferation and migration may be partly associated with an increased level of Erk phosphorylation [18, 19]. To explore the possible mechanism of GSPP in inhibiting bFGF-induced lymphangiogenesis, Erk and p-Erk protein expressions were detected by western blotting. Results showed that bFGF significantly increased the expression of p-Erk in hLECs and GSPP decreased the bFGF-induced p-Erk significantly. No significant difference of the expression of total Erk was observed among each group (Figure 1(c)).

### 3.4. GSPP Inhibited bFGF-Induced Migration of hLECs

**Figure 2:**
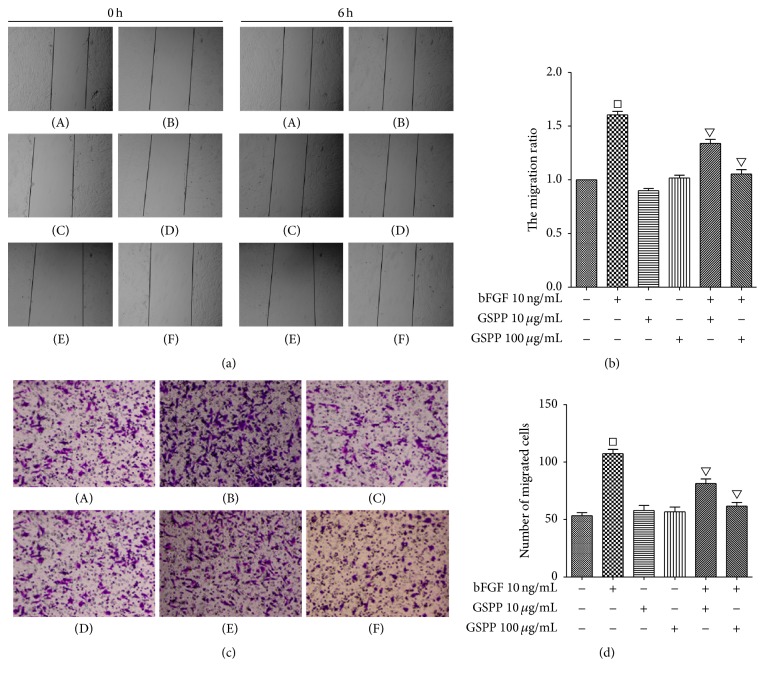
GSPP inhibits bFGF-induced migration of hLECs. (a) Wound healing assay. Left, representative images of injury width in wound healing assay (×40). Right, quantification of the migration ratio of hLECs compared to control. hLECs (1 × 10^5^ cells/well) were seeded in 24-well plates and wounds were generated after cell confluence. After hLECs were treated with different concentrations of GSPP (0, 10, and 100 *μ*g/mL) and/or bFGF (10 ng/mL) for 0 h and 6 h, the photos were taken and the injury width was measured. The migration ratio was calculated as the migration width of experiment group/the migration width of control group. (b) Transwell assays. Left, representative images of migrated LECs in transwell assay (×40). Right, quantification of migrated LECs compared to control. hLECs (5 × 10^4^ cells/well) in EBM-2 with different concentrations of GSPP (0, 10, and 100 *μ*g/mL) were added to the upper chamber of the transwell insert. EBM-2 containing bFGF (10 ng/mL) or not was added to the lower chamber to induce cell migration. After 12 h at 37°C, cells on the top surface of the membranes were wiped off with cotton balls, and the cells that migrated on the underside of inserts were fixed with methanol and stained with crystal violet. Five different digital images were taken per well, and the number of migrated cells was counted. (A) Control, (B) bFGF 10 ng/mL, (C) GSPP 10 *µ*g/mL, (D) GSPP 100 *µ*g/mL, (E) GSPP 10 *µ*g/mL + bFGF 10 ng/mL, and (F) GSPP 100 *µ*g/mL + bFGF 10 ng/mL. Data were presented as mean ± SD of three independent experiments; ^□^ *P* < 0.05 versus control group and ^▽^ *P* < 0.05 versus bFGF-single use group.

Wound healing experiment and transwell experiment were conducted to investigate the antimigration effect of GSPP. It was showed that bFGF significantly upregulated the migration distance of hLECs (*P* < 0.05), which was antagonized by GSPP significantly (*P* < 0.05) at 6 h (Figure 2(a)). The same results were obtained in transwell experiment. The number of migrated cells in bFGF-treated group increased significantly compared to the negative control cells (*P* < 0.05). No significant difference of the cell numbers was observed between the GSPP-treated group and negative control (*P* > 0.05). However, concomitant treatment with 10 *μ*g/mL, 100 *μ*g/mL GSPP, and bFGF inhibited the migration of hLECs compared with the bFGF-single use group (*P* < 0.05) (Figure 2(b)).

### 3.5. GSPP Abrogated bFGF-Induced hLECs Lymphangiogenesis In Vitro

**Figure 3:**
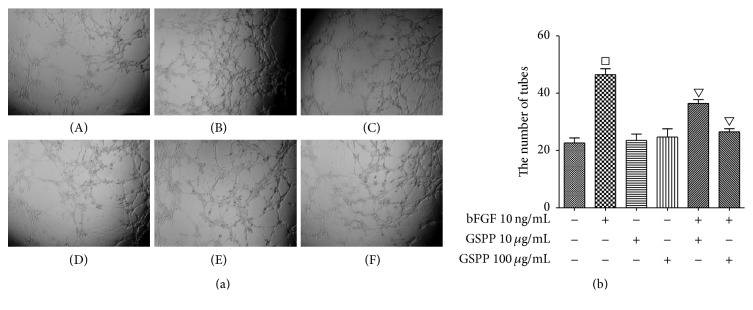
GSPP inhibits bFGF-induced lymphangiogenesis in vitro. In vitro tube formation assay. hLECs (1.5 × 10^4^ cells/well) were seeded in Matrigel-coated 96-well plates and treated with different concentration of GSPP (0, 10, and 100 *μ*g/mL) and/or bFGF (10 ng/mL) for 4 h and the tube-like structure formation was observed. (a) Representative images of tube formation (×40). (b) Quantification of inhibitory ratios of tube branches. (A) Control, (B) bFGF 10 ng/mL, (C) GSPP 10 *µ*g/mL, (D) GSPP 100 *µ*g/mL, (E) GSPP 10 *µ*g/mL + bFGF 10 ng/mL, and (F) GSPP 100 *µ*g/mL + bFGF 10 ng/mL. Data were presented as mean ± SD of three independent experiments; ^□^ *P* < 0.05 versus control group and ^▽^ *P* < 0.05 versus bFGF-single use group.

Tube-like formation assay was conducted to examine the inhibitory effect of GSPP on lymphangiogenesis. hLECs were added on top of the gel in the 96-well plate, incubated at 37°C in a tissue culture incubator, and the formation of the capillary-like tubes was observed after 4 h. There was no difference in the number of tube-like structures formation between GSPP treatment group and the negative control group in hLECs (*P* > 0.05). The number of tube-like structures treated with bFGF group was much more than the negative control group (*P* < 0.05). However, GSPP significantly attenuated bFGF-induced tube formation in a dose-dependent manner with simultaneous incubation with GSPP and bFGF (Figures 3(a) and 3(b)).

### 3.6. GSPP Inhibited Tumor Growth and Lymphangiogenesis in HT-29 Colon Carcinoma Nude Mice Model

**Figure 4:**
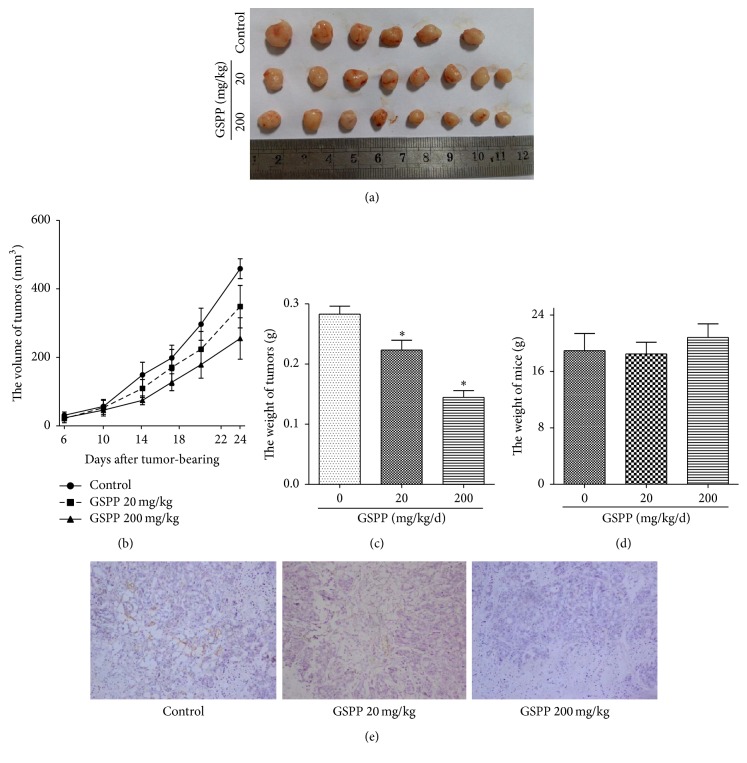
GSPP inhibited colon carcinoma HT-29 xenograft growth and lymphangiogenesis in vivo. Male nu/nu nude mice were inoculated subcutaneously with colon carcinoma HT-29 cells. Three days after inoculation, mice were treated with GSPP (20 or 200 mg/kg) or PBS every day for 21 days via intraperitoneal injection. The lengths and widths of tumors were measured individually every 3 days. At the end of the experiment, the implanted tumors were sectioned. (a) Effect of GSPP on tumor volume. Left, image of excised tumors. Right, tumor growth curves. (b) Effect of GSPP on tumor weight. (c) Effect of GSPP on mouse weight. Mice were weighed at the end of the experiment. (d) Tumor lymphatic microvessel density. The implanted tumors were sectioned and stained against LYNE-1 antibody. Tumor lymphatic vessels are shown as LYNE-1 positive (yellow color). Data were presented as mean ± SD of three independent experiments; ^*∗*^ *P* < 0.05 versus control group.

The antitumor activity of GSPP was investigated in HT-29 colon carcinoma xenograft model using BALB/c nude mice. Growth of the tumors was significantly inhibited in the mice treated with GSPP compared with the growth of tumors in control mice (Figure 4(a)). In control group (intraperitoneal injection with PBS), tumors grew rapidly and reached an average volume of 459.03 ± 28.92 mm^3^ (mean ± SD) by day 24 after being transplanted with HT-29 tumor blocks, while the sizes of tumors in 20 and 200 mg/kg/day GSPP-treated groups were only 348.25 ± 62.2 mm^3^ and 255.18 ± 60.72 mm^3^, respectively (75.9% and 55.9% decrease) (*P* < 0.01, Figure 4(b)). The tumor weight of the control group was 0.28 ± 0.03 g, whereas the weights of GSPP-treated groups decreased to 0.22 ± 0.04 g and 0.14 ± 0.03 g, respectively (*P* < 0.01, Figure 4(c)). To evaluate the adverse effects of GSPP, we measured the weights and visceral index of the mice and found that there was no significant difference between the control and GSPP-treated groups (Figure 4(d)). Tumor lymphangiogenesis was analyzed using immunohistochemical staining with LYVE-1 antibody. Results showed that 200 mg/kg/day GSPP markedly reduced tumor microvessel density in the tissue sections compared with the control group (Figure 4(e)). These results indicated that GSPP efficiently inhibits tumor growth in carcinoma animal model. Suppression of tumor growth due to GSPP could be caused by inhibition of lymphangiogenesis.

## 4. Discussion

Metastasis is a key cause for the failure of tumor treatment and patient death. The lymph node metastasis is the first step of the tumor dissemination and is also the main sign of poor prognosis of tumor [20–22]. More and more studies showed that the tumor-induced lymphangiogenesis played an extremely important role in cancer cells spreading to some local lymph nodes and distant metastasis [23–25]. Studies on antitumor lymphangiogenesis have gradually become a research hotspot, and bFGF as an important factor to promote lymphangiogenesis has been researched intensively.

*Gekko swinhonis *Günther was a traditional Chinese medicine, which has been used as an anticancer drug in traditional Chinese medicine for hundreds of years, especially in hepatoma [26]. Soaking in alcohol is very useful for pharmaceutical ingredients dissolving out and is also the most common method of extraction. Oral administration and external use were the main methods of administration. In our previous study, we isolated GSPP from* Gekko swinhonis *Günther and confirmed that GSPP could induce hepatoma cell differentiation, inhibit cell proliferation and migration in vitro, and inhibit hepatic carcinoma growth in vivo [12, 15, 17]. In this study, we verified another mechanism of GSPP's antitumor effects by inhibiting tumor lymphangiogenesis. Results showed that GSPP significantly inhibited bFGF-induced human hLECs proliferation, migration, and tube-like structure formation in vitro. Moreover, GSPP treatment (200 mg/kg/d) not only inhibited the growth of breast carcinoma, but also inhibited the lymphangiogenesis in vivo. The dosage was determined by previous studies, which is a safe dose with an appropriate tumor inhibition rate [12]. Through this study, we further uncovered the mechanisms of GSPP's antitumor effects besides inhibiting cancer cells proliferation and migration, inducing tumor cell differentiation, and inhibiting cancer angiogenesis and cancer-associated fibroblast growth (unpublished), showing that GSPP is a promising antitumor drug in future cancer treatment.

bFGF, as an important prolymphangiogenesis factor generated by tumor cells, can significantly promote lymphatic vessel endothelial cell proliferation and migration and promote tumor lymphangiogenesis by a variety of ways [27]. The most classic way on which bFGF promotes tumor lymphangiogenesis is through VEGF-A, VEGF-C, and VEGF-D, which are known prolymphangiogenesis factors to promote tumor lymphatic vessel grow [23, 28]. bFGF is a heparin dependent growth factor, which means that it can have effect only in the form of bFGF-heparin-FGFR terpolymers structure to further activate the intracellular signal transduction pathways. GSPP is a kind of polysaccharide sulfate, which is similar to the structure of heparin active site, both of which contain a sulfuric acid base. Previous study showed that GSPP works by three distinct mechanisms: (a) blocking the bFGF production, (b) inhibiting the release of bFGF from the extracellular matrix, and (c) directly binding to bFGF and competitively inhibiting the binding of bFGF to its low affinity receptor heparin/HS [12]. In this study, we found that GSPP alone has no effect on the viability, growth of hLECs. And it does not inhibit the migration and tube formation of hLECs, which may be due to the absence of bFGF. bFGF plays an important role in the growth, migration, and lymphangiogenesis of hLECs. With the addition of exogenous bFGF, which simulates the environment in the body, GSPP significantly inhibited bFGF-induced cell proliferation, migration, and tube formation. This means that GSPP works through directly binding to bFGF and competitively inhibiting the binding of bFGF to its low affinity receptor heparin/HS.

Extracellular signal-regulated kinase1/2 (Erk1/2) is a protein kinase, separated and identified in the early 1990s, and its signal transduction is involved in cell growth, development, and differentiation [19]. Studies found that the promoting effect of bFGF on endothelial cell proliferation and migration of part is associated with an increased level of Erk phosphorylation [18, 29]. To explore the possible mechanism of GSPP's inhibiting effect of bFGF-induced lymphangiogenesis, western blot was performed. We examined the Erk and p-Erk expression level changes in lymphatic endothelial cells after being exposed to GSPP for a certain time. Our results showed that bFGF significantly increased the expression of p-Erk in hLECs. However, cotreatment with GSPP and bFGF decreased the expression of p-Erk in hLECs compared with the bFGF-single use group. This further proved that GSPP could bind to bFGF and competitively inhibit the binding of bFGF to its low affinity receptor, thus blocking the bFGF-induced phosphorylation of Erk1/2 in hLECs.

## 5. Conclusions

Our study showed that inhibiting bFGF-induced lymphangiogenesis was one of GSPP's anticancer mechanisms. GSPP, as an effective bFGF-targeted inhibitor, can be a notable antilymphatic metastasis drug in future cancer treatment.
